# Gaussian Process Regression for Tail Vehicle Departure Time Prediction at Signalized Intersections Using UAV Trajectory Data

**DOI:** 10.3390/s26113364

**Published:** 2026-05-26

**Authors:** Kaiming Lu, Zhe Liu, Runsheng Zhang, Qingyang Xia, Ruoxuan Wang

**Affiliations:** Transport Planning and Research Institute, Ministry of Transport, Beijing 100028, China; lukaiming@tpri.org.cn (K.L.); liuzhe@tpri.org.cn (Z.L.); xiaqy@tpri.org.cn (Q.X.); wangrx@tpri.org.cn (R.W.)

**Keywords:** vehicle departure time, machine learning, Gauss process regression, traffic uncertainties, multi-lane signalized intersections

## Abstract

Extensive research has been conducted on vehicle queuing and dissipation near signalized intersections. However, existing prediction methods for vehicle departure time primarily rely on assumptions of steady-state homogeneous traffic flow, utilizing shockwave theory and vehicle kinematic modeling. These methods encounter challenges in addressing traffic uncertainties during queue formation and dissipation, particularly in scenarios involving multiple lanes. This paper introduces a novel approach by leveraging unmanned aerial vehicle (UAV) trajectory data to construct fleet state features and proposes a prediction method for tail vehicle departure time based on Gaussian process regression. The objective of this method is to optimize the green light crossing time window and eco-driving trajectory for connected vehicles at signalized intersections. The findings reveal that the departure time of the tail vehicle within a specified distance adheres to a Gaussian process, demonstrating the applicability of Gaussian process regression for departure time prediction modeling. The effectiveness of the proposed method was validated using a field-measured dataset collected from three typical multi-lane signalized intersections. Notably, compared to four benchmark models (linear regression, decision trees, multilayer perceptron neural networks, and eXtreme Gradient Boosting—XGBoost), the mean absolute percentage error (MAPE) was reduced by an average of 5.146% on the test set under a random 70/30 split. Additionally, a robustness assessment demonstrates that the proposed model performs well, albeit slightly less effectively than the XGBoost model. We emphasize that the conclusions are drawn for the studied intersections; generalization to unseen intersections requires further validation with cross-site data.

## 1. Introduction

The reliable prediction of queue dissipation time at signalized intersections is a critical constraint for trajectory planning and control in connected and automated driving systems. However, the departure time of the trailing vehicle from the stop line exhibits uncertainty when vehicles are not fully queued, posing significant challenges for accurate prediction. Correspondingly, vehicle queue delay, as a component of departure time, has been recognized as an important traffic performance measure and has been studied extensively using various estimation models, such as deterministic queueing models, shockwave models, and steady-state stochastic delay models [1,2]. These methods typically require the prior estimation of queue length and are primarily used for signal control and evaluation. For the eco-driving control of vehicles, it is essential to predict the time required for any vehicle (the host vehicle) with an opportunity to engage in eco-driving to completely clear the intersection, thereby determining whether the host vehicle can traverse the signalized intersection within the green light window and at what speed trajectory.

As mentioned above, research utilizing queue length to indirectly predict delay reflects certain characteristics: it necessitates assuming that traffic flow conforms to a steady-state homogeneous traffic flow, which diverges from actual traffic conditions and fails to capture traffic uncertainties. Due to the heterogeneous nature of driving behaviors during queue formation and dissipation, including factors like reaction time and acceleration, the relationship between queue length and dissipation time is not purely linear. Consequently, many studies have explored using machine learning methods to predict queue length or dissipation time [3,4,5,6], primarily relying on low-penetration floating vehicle GPS trajectory data or sectional traffic flow detection data. Due to limitations in traffic perception conditions, these methods still rely on micro or macro traffic flow models to make uncertain estimates. However, with advancements in intelligent perception devices and vehicle-to-infrastructure communication technology, research on departure time (equivalent to queue dissipation time when all vehicles in the queue are waiting) based on novel sensing technologies may leverage machine learning methods to capture the nonlinear relationship between departure time and various influencing factors, thereby effectively addressing traffic uncertainties and achieving high-precision departure time prediction.

However, existing studies on queue dissipation predominantly assume homogeneous traffic flow and fully formed stationary queues, leaving the prediction of the last vehicle’s departure time—especially under uncertain or non-stationary queue formation—largely unaddressed. To fill this gap, this paper proposes a data-driven method to predict the departure time of the last vehicle using drone-measured data. The primary contributions are as follows:

1. This study leverages the advantages of Gaussian process regression (GPR) to address traffic uncertainties and develops a departure time prediction model based on GPR for tail vehicle departure time at signalized intersections.
2. Empirical data from the studied intersections is used to validate the applicability and rationality of the GPR-based approach for departure time prediction under a random 70/30 training–test split.
3. Comparative experiments are conducted between the GPR-based method and several commonly used machine learning methods, demonstrating the performance advantages of the GPR model within the same intersections and assessing the differences in robustness among the various models.

The rest of this paper is organized as follows. In Section 2, the literature review is introduced. Section 3 states the problem to be discussed. Section 4 details the methodology proposed in this paper. Section 5 presents the experiments results and conducts comparisons between various models using field-measured data. Section 6 concludes this paper and discusses future research directions.

## 2. Literature Review

Under congested traffic conditions, tail vehicle departure time at signalized intersections is closely related to queue dissipation characteristics, queue length, and other factors. However, most existing studies have focused on methods for estimating or predicting these parameters while neglecting the prediction of vehicle departure times from the stop line—an aspect of great significance for eco-driving trajectory planning and control. Therefore, this section first reviews the methods for estimating or predicting traffic parameters related to queueing at intersections, then focuses on the existing research approaches for predicting vehicle departure times from the stop line in the context of eco-driving trajectory planning. Finally, it summarizes the limitations of current research methods.

### 2.1. Queue Dissipation Characteristics

Queue dissipation characteristics have long been an important component of studies on traffic flow theory at signalized intersections. As early as 1979, G. Stephanopoulos investigated the impact of signal control parameters such as signal cycle length and green and red intervals, as well as system parameters including arrival rates and capacity, on queue lengths [7]. Zhuang-zhi, S. et al. explored the relationship between the startup time of the leading vehicle in a queue and traffic interference at signalized intersections [8]. They applied logistic, Gompertz, and logistic models to study the discharge headway of vehicles in left-turning, inside through, and outside through lanes, respectively, demonstrating significant improvement in predictive accuracy compared to traditional models. An, C. et al. proposed a vehicle discharge process modeling method based on Hidden Markov Models (HMMs), facilitating vehicle discharge state identification using continuous travel time and discharge headway observations as inputs, encoding three states: overflow, single stop, and free arrival [9]. Bhattacharyya, K. et al. introduced a new metric called the “vehicle passing interval” for non-lane-based traffic, which effectively captured the heterogeneity of vehicle types and discharge rates [10]. Analyzing empirical data, Al-kaissi et al. discovered that concentrated distributions of smaller headways pose higher potential risks [11]. Studies by Mondal, S. et al. and Sharma, A. et al. investigated the effects of signal light durations and countdown timers on queue dissipation, highlighting their significant impact on headway distributions and the reduction in startup losses [12]. Sharma et al., using actual data from video recordings of two intersections in India, analyzed queue dissipation characteristics under mixed traffic conditions with and without countdown timers [13]. The results indicated that countdown timers significantly affect the distribution of headways and are beneficial in reducing startup loss time.

Additionally, Limanond et al. discussed the level of improvement in intersection capacity due to countdown timers [14]. For mixed traffic flow, Dey et al. analyzed measured data to demonstrate that as queues dissipate, headways compress, albeit differently from saturated traffic flow [15]. They also examined the clearing rates of vehicles at different queue positions and the changing trends in intersection crossing times. Mondal et al. further studied the distribution characteristics of dissipation headways in mixed traffic with interference, conducting headway analysis using queue dissipation data from 20 intersections and comparing optimal fitting functions for headway distributions at different queue positions [16]. Moreover, vehicle kinematic models and traffic shockwave theories are commonly used methods for analyzing queue dissipation characteristics. For instance, Qu et al. analyzed variations in the dissipation process of leading vehicles, the time taken for following vehicles to reach steady-state speeds, and the relationship between green signal duration and dissipation vehicle count [17]. However, to address the limitations of existing queue dissipation models, which heavily rely on macroscopic traffic flow or simplistic assumptions that fail to accurately represent real dissipation characteristics, Srivastava et al. proposed an improved Cell Transmission Model (CTM) by modifying the traditional demand function [2]. This new demand function integrates critical parameters like critical density, free-flow speed, jam density, and an additional parameter, jam demand. Model calibration and simulation analysis confirmed the model’s ability to generate realistic queue dissipation features. Looking towards future advanced transportation systems, Wei Xu et al. studied an enhanced optimal velocity (OV) model in a V2X environment, considering drivers’ early response times, and analyzed the impact of different initial headways, maximum speeds, and safety distances on intersection traffic flow [18]. They used intersection queue dissipation efficiency (or queue dissipation rate) as an evaluation metric for these impacts.

### 2.2. Queue Length Estimation

Queue length estimation is a crucial metric for assessing and optimizing intersection operations, leading to a wealth of research outcomes. For instance, Bezuidenhout, J. et al. proposed a novel queue estimation model based on data collected from stop line loop detectors at signalized intersections, distinguishing between queued and non-queued vehicles to indirectly estimate potential queue lengths [19]. Given that traditional deterministic queueing theories are based on the assumption of stable traffic flow throughout, which does not align with real-world conditions, Yu, L. et al. modeled queue dynamics at signalized intersections using the Lighthill–Whitham–Richards (LWR) shockwave theory instead of deterministic queueing theories [20]. Zhan et al. developed a Gaussian process-based cumulative arrival–dissipation curve prediction model, achieving the indirect prediction of lane-level maximum queue lengths and validating the model’s effectiveness using license plate recognition data [4]. Additionally, Mei et al. analyzed variations in vehicle queue lengths over multiple signal cycles using low-penetration global position system (GPS) trajectory data, constructing a Bayesian estimation-based model for cycle-level maximum queue length estimation [3]. Recently, with the development of V2X communication technology and roadside lidar data application, new queue length estimation methods were proposed [21,22].

### 2.3. Estimation of Traffic Parameters Related to Queue Dissipation

Apart from queue length, other traffic parameters related to queue dissipation have also garnered significant attention. For instance, Teng et al. proposed a vehicle delay estimation algorithm based on vehicle GPS trajectory data, strictly adhering to data extraction during stages such as vehicle slowing down at signalized intersections, waiting in the queue, waiting for discharge, and discharge [23]. However, this work is solely intended for the later evaluation of intersection operational quality, serving traffic management, and does not involve delay prediction tasks. Lu et al. studied individual vehicle travel time prediction models on arterial roads, dividing travel time into intersection delays and basic segment travel times, with a particular focus on constructing an intersection delay estimation model based on shockwave theory [24]. Nevertheless, this model is primarily applicable to single-lane scenarios. Focused on predicting signalized intersection traffic states, Sun et al. developed a short-term vehicle speed prediction method based on V2X communication technology sensing information, constructing the method using a modified second-order Payne–Whitham (PW) model to reflect signal influences on vehicles/traffic flow, with research showing improved speed prediction accuracy [25]. Guchang et al. introduced a wavelet–Elman neural network-based approach to predict the time when the lead vehicle arrives at an intersection, defined as the time difference from when the red light comes on to when the vehicle reaches the stop line, which aids in extracting delay variables and facilitates signal timing optimization [26]. Additionally, Fang et al. proposed a model to estimate traffic flow at the stop line using upstream loop detector traffic information, considering vehicle dynamics, arrival rates, and departure rates and incorporating different vehicle speeds, queue lengths, and signal timing parameters [27].

### 2.4. Intersection Eco-Driving Considering Traffic Constraints

Eco-driving at signalized intersections is a significant research topic, with traffic spatiotemporal constraints being crucial factors in planning or controlling eco-driving trajectories. For instance, Xin et al. introduced a predictive intelligent driver model (IDM) with forecasting capabilities, considering signal lights and queue dissipation times as forward traffic spatiotemporal constraints to avoid vehicle idling and stops [28]. Chao Sun et al. considered the uncertainty of signal timing, proposing an eco-driving robust optimization control model for signalized intersections [29]; however, queue dissipation effects were not considered, which are inevitable in real traffic environments. Fei Ye et al. proposed an eco-approach and departure (EAD) strategy considering short-term speed predictions of preceding vehicles, estimating queue length and delay [30]. Given that dynamic changes in queue at signalized intersections may impede the effective execution of eco-driving trajectories for connected vehicles due to safety concerns, modeling and predicting the waiting queue’s dynamic variation based on shockwave theory and data-driven traffic flow prediction are essential [29]. Hao Yang et al. proposed an Eco-Cooperative Adaptive Cruise Control strategy considering deterministic queue lengths, estimating the time the last vehicle in the queue leaves the stop line using shockwave theory [31]. Dong proposed a hierarchical energy-efficient control strategy for connected vehicle eco-driving, considering queue discharge times based on theoretical estimation using kinematic models, primarily targeting single-lane stationary queue scenarios, which may not adapt well to traffic uncertainties [32]. Additionally, Dong also proposed a hierarchical energy-efficient control strategy considering spatial and temporal constraints from preceding vehicles and queues [33].

### 2.5. The Existing Research Limitations

From the above review, it is evident that previous research has mainly focused on studying queue dissipation characteristics, queue length estimation, and other traffic parameter estimations such as delays. Although some studies related to signalized intersection eco-driving consider queue dissipation times or the departure time of the last vehicle, there are still shortcomings. Firstly, the estimation/prediction methods of these time constraints mainly rely on macroscopic traffic flow fundamental diagram models or vehicle kinematic models, all based on the assumption of homogeneous vehicle arrival, departure, and homogeneous traffic flow. However, this may not align with actual traffic attributes, so predicting departure times based on steady-state homogeneous traffic flow assumptions cannot accommodate the diverse forms and patterns of vehicle queue formation and dissipation, which may compromise prediction accuracy. Secondly, most studies have focused on scenarios near intersections with stationary queues; however, in reality, at the end of the red light or green light periods, stationary queues may not necessarily form within a certain range of signalized intersections, and vehicle spatial distribution exhibits randomness. Therefore, there is currently a lack of departure time prediction methods suitable for such uncertain scenarios. We understand that linear regression was used to calibrate functional relationships between total queue dissipation duration and key contributing variables, including passenger cars, sport utility vehicles or trucks, heavy vehicles, and three binary variables [34]. Additionally, Chen et al. employed deep learning methods to capture queue characteristics from video and estimate queue dissipation times [6]. However, these methods are also applicable only in scenarios where stationary queues exist after the green light onset. Hence, constructing a unified vehicle departure time prediction model considering the uncertainty of traffic states near intersections is of significant practical importance for eco-driving trajectory planning. To address this, this paper utilizes drone-measured data, employing supervised learning algorithms to construct a vehicle departure time prediction model, and validates its effectiveness, applicability, and performance advantages.

To present the current research status and limitations in this field more clearly, a summary is provided in Table 1 considering four aspects: research area, representative studies, methodology/data, and key limitations or unresolved issues.

## 3. Problem Statement

**Figure 1:**
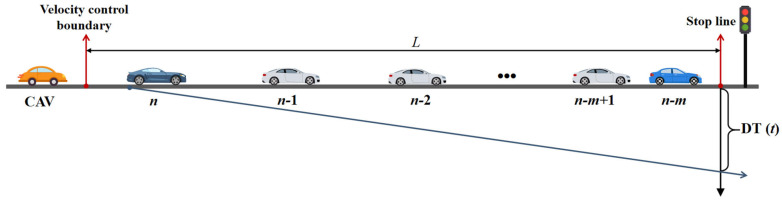
Diagram of research scenario.

This study is aiming at predicting the departure time (DT) of the tail vehicle in a fleet within a specified spatial range (*L*, shown in Figure 1) at a signalized intersection. This prediction serves to aid in trajectory planning and control for connected and automated vehicles (CAVs) within an eco-driving framework. As illustrated in Figure 1, when a CAV approaches the eco-driving control boundary at time *t*, it is necessary to forecast the time DT(*t*) until the clearance of preceding vehicles, enabling the derivation of an eco-trajectory for CAVs.

However, the queueing and dissipation process of vehicles ahead of CAVs exhibit considerable uncertainty, posing challenges for accurately predicting DT. Traditional theory-based models may struggle to adapt to traffic uncertainties. Therefore, this study aims to propose a machine learning approach for predicting DT to enhance prediction accuracy and ensure adaptability to complex traffic environments. Notably, due to the close relationship between DT and signal light states, this paper focuses on predicting DT under two specific scenarios: when the current signal status is red with less than 15 s remaining (but data with up to 20 s remaining are also included for distribution validation purposes) and when the signal is green. The 15 s threshold is based on empirical observation from our field data: vehicles that arrive when the remaining red time exceeds 15 s generally do not pass the stop line during the same cycle, making the prediction of the tail vehicle departure time less meaningful for real-time eco-driving applications. Therefore, this threshold ensures that the prediction task focuses on scenarios where the last vehicle’s departure time is directly relevant.

## 4. Methodology

In this section, we focus on introducing the prediction method for the departure time (DT) of the tail vehicle in a fleet. Specifically, GPR, a supervised machine learning method, was used to predict DT. A Gaussian process (GP) is defined as a random process consisting of infinite random variables following a Gaussian distribution (GD) in a continuous time or spatial domain [35]. It has been applied in the fields of traffic flow prediction and airport capacity prediction [36,37]. In this paper, the predicted DT within the control range can be considered a random variable and is assumed to follow a GD. In a continuous time domain, such as a period of one signal cycle (the vehicle trajectories of multiple consecutive signal cycles can be projected into one signal cycle for a fixed-timing signalized intersection), if a set of predicted DTs at an arbitrary moment follows a high-dimensional GD, then the function of DT within this cycle can be regarded as a GP. In addition, the departure velocity of the subject vehicle is considered a random variable that is correlated with the preceding traffic state.

### 4.1. GPR Fundamentals

Given a training set containing n input and output pairs, where an arbitrary element $x_{i}$ of the input set $X={\left[x_{1},x_{2},\ldots ,x_{n}\right]}^{T}$ is a *D*-dimensional vector, i.e., $x_{i}\in {\mathbb{R}}^{D}$, the training input $x_{i}$ is related to a scalar value $y_{i}$, which is one of the output vectors. The observed departure time values are conditioned on the latent function values with Gaussian noise $\varepsilon_{i}$; $y_{i}=f_{i}+\varepsilon_{i}$, where $\varepsilon_{i}\sim N(0,\sigma_{\varepsilon }^{2})$ is the noise, and the n latent variables are collected into a vector form $f={\left[f_{1},f_{2},\ldots ,f_{n}\right]}^{T}$. We assume that a random vector $f_{i}$ of all latent function values follows a GP, which can be specified as

(1) $$f_{i}\sim GP(\mu (x_{i}),k(x_{i},x_{j})),i,j=1,2,\ldots ,n,$$

where μ(⋅) is a predefined mean function, and k(⋅,⋅) is the covariance/kernel function. Different kernel functions can be used to generate a valid covariance matrix. In this work, we set μ(⋅)=0 and use the automatic relevance determination (ARD) squared exponential function as the covariance kernel; this is the squared exponential kernel function with a separate length scale $\sigma_{m}$ for each predictor m (*m* = 1, 2, …, *d*). It is defined as

(2) $$k(x_{i},x_{j}|\theta )=\sigma_{f}^{2}\exp\left[-\frac{1}{2}\sum_{m=1}^{d}\frac{{\left(x_{im}-x_{jm}\right)}^{2}}{\sigma_{m}^{2}}\right],$$

where $\sigma_{f}^{2}$ is the signal standard deviation, and $\sigma_{m}$ is a d-dimensional vector of length scales. These kernel parameters are normally referred to as hyperparameters. The unconstrained parametrization θ in this case is

(3) $$\theta_{m}=\log\sigma_{m},\mathrm{for}m=1,2,\ldots ,d,$$

(4) $$\theta_{d+1}=\log\sigma_{f}.$$

This kernel function is an adaptive correlation function that considers the degree of influence of each feature on the prediction by adjusting the correlation coefficient of each feature automatically according to the data characteristics. Thus, it helps improve the prediction accuracy and stability of the model.

Finally, the recombined vector of the observed target values *y* and the function values $f^{*}$ at the test locations follows a joint GD and can be written as

(5) $$\left[\begin{array}{l} y\\ f^{*} \end{array}\right]\sim N\left(0,\left[\begin{array}{cc} K(X,X)+\sigma_{\varepsilon }^{2}I & K(X,X^{*})\\ K(X^{*},X) & K(X^{*},X^{*}) \end{array}\right]\right),$$

where $K(X,X^{*})$ denotes the covariance matrix evaluated at all pairs of *n* training points and $n^{*}$ test points.

By conditioning the joint Gaussian prior distribution on the observations, we can derive the joint posterior distribution over the function values and obtain the predictive models:

(6) $$p(f^{*}|X,y,X^{*})\sim N({\bar{f}}^{*},cov(f^{*}))$$

where

(7) $${\bar{f}}^{*}=K(X^{*},X){\left[K(X,X)+\sigma_{\varepsilon }^{2}I\right]}^{-1}y$$

(8) $$cov(f^{*})=K(X^{*},X^{*})-K(X^{*},X){\left[K(X,X)+\sigma_{\varepsilon }^{2}I\right]}^{-1}K(X,X^{*})$$

Before prediction, the hyperparameters should be optimized to build the kernel function. Bayesian inference can be used to estimate the parameters of the GP model. The model parameter vector θ consists of $\sigma_{f}^{2}$, $\sigma_{m}$, and $\sigma_{\varepsilon }^{2}$, and it can be estimated by maximizing its log posterior probability:

(9) $$\hat{\theta }=\arg\max\left\{\log p(\theta |X,y)\right\}=\underset{\theta }{\arg\max}\left\{\log p(\theta )-\frac{1}{2}y^{T}K_{y}^{-1}y-\frac{1}{2}\log|K_{y}|-\frac{n}{2}\log2\pi \right\},$$

where p(θ) is the prior distribution over the hyperparameters, n is the number of training observations, y is the vector of observed output values, and $K_{y}$ is the covariance matrix obtained by applying the kernel function over pairs of input vectors in X.

### 4.2. Feature Construction

Previous studies on driving behaviors at signalized intersections revealed that signal status, the velocity of preceding vehicles and distance from the stop line are the key influences on individual driving behaviors [38,39]. In addition, the micro driving behavior parameters of startup time and stopping spacing are introduced to predict the departure time of waiting queues [32]. Inspired by these works, we preliminarily selected 8 statistical features of macro traffic flow, which are easy to detect and calculate, as the inputs of the GPR-based departure time prediction model. They are specified in Table 2.

To select features highly correlated with vehicle departure time and reduce the complexity of the GPR-based prediction model, we use the widely used methods of the Pearson correlation coefficient and an interpretable machine learning framework (SHapley Additive explanation, SHAP) to analyze the correlation between features and choose key features eventually. The Pearson correlation coefficient can reflect the linear correlations among features only and fails to reflect nonlinear relationships. Meanwhile, SHAP can be used to copy complex nonlinear problems and analyze feature importances from the perspective of individual samples. This approach is widely adopted to mine and predict traffic characteristics using big data, especially the identification and selection of key features [40,41,42,43]. Note that SHAP should be based on a machine learning model (e.g., tree models) that can be explained. In this study, we choose the eXtreme Gradient Boosting (XGboost) algorithm to build the prediction model.

### 4.3. Feature and Label Extraction Algorism

**Figure 2:**
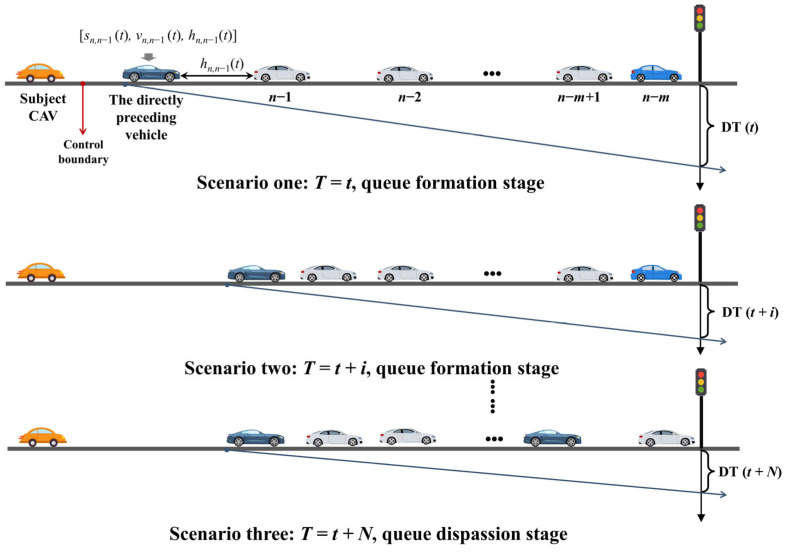
An illustration of the random process of the departure time DT(t) within the control range.

As shown in Figure 2, when a CAV reaches the control boundary, it will encounter different traffic states characterized by parameters such as the average speed and average spacing of preceding vehicles. The traffic state will change over time, and each state at moment t will correspond to an observed value of departure time in the future, namely, DT(*t*). As mentioned in Section 4.1, if it is assumed that DT(*t*) follows a GD at any moment, then the set of DT(T)=[DT(t),DT(t+1),…,D(t+N)],T∈[t,t+N] could be considered a GP. The feature and label extraction algorithm of vehicle departure time is shown as Algorithm 1.
**Algorithm 1:** Feature and Label Extraction of Vehicle Departure Time**Input:** *L*: Spatial range from the stop line for monitoring; Signal state and remaining red light time; Vehicle information (position, speed, etc.) in the straight lane. **Output:** DT(*Ts*): Departure time sample for the tail vehicle at each timestamp *Ts*. 1: **Function:** LableExtraction (*L*, signal_state, remaining_red_time, vehicle_info) 2:        **Initialization:** 3:        Set the spatial range to *L* from the stop line. 4:        Initialize the recording start time *Ts* as 0. 5:        Initialize the loop count and loop interval. 6:        **Start Loop:** 7:             **Detect Signal State:** 8:                      Check the current signal_state and remaining_red_time. 9:             **If** (the signal_state is red **and** remaining_red_time <= 15 s) **or** the signal_state is green: 10:                      Record the current time as *Ts*. 11:                      Extract the information (position, speed, etc.) of each vehicle in the straight lane from vehicle_info at *Ts*. 12:                      Number the vehicles from front to back (1, 2, 3, …, *n*−1). 13:                      Calculate the operational status feature variables of the vehicle platoon (average speed, average inter-vehicle distance, etc.). 14:                      **Check** whether the tail vehicle (vehicle number *n*) changes lanes or not. 15:                      **If** the tail vehicle changes lanes: 16:                               Discard the current sample. 17:                      **Else:** 18:                               Record the time *T_out* when vehicle *n* completely crosses the stop line. 19:                               Calculate the departure time sample DT(*Ts*) for vehicle *n*, DT(*Ts*) = *T_out* − *Ts*. 20:             **End If** 21:             Wait for 1 s. 22:             Update *Ts* to *Ts* + 1 s. 23:             **Continue** to the next iteration. 24:        **End Loop** 25:        **Return:** DT(*Ts*) for each timestamp *Ts*. 26: **End Function**

## 5. Experiments and Results

### 5.1. Dataset Collection and Processing

The field-measured data was acquired using UAVs in an actual traffic setting at three fixed-timing signalized intersections in Yanjiao, Hebei Province, China. The UAV operated at an altitude of 200 m, covering approximately 270 m along the road direction. Video footage was captured at a framerate of 30 Hz, spanning a cumulative recording duration of approximately 4 h. The flight sessions were conducted during three distinct periods: morning peak (7:00–9:00), evening peak (17:30–19:00), and off-peak (14:00–16:00) hours. Simultaneously, signal timing plans were collected during these operations.

**Figure 3:**
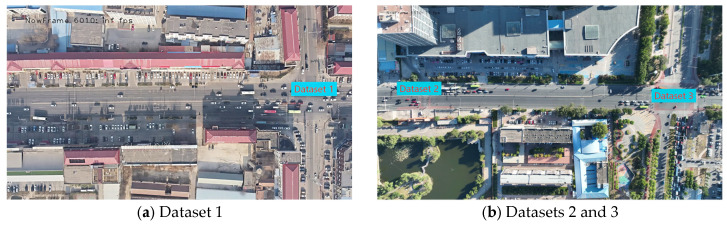
Dataset collection scenes.

The first intersection entrance features four lanes: a dedicated left-turn lane, two through lanes, and a right-turn lane. The second intersection consists of three through lanes and one right-turn lane. At the third intersection approach, there are two dedicated left-turn lanes, two through lanes, and one right-turn lane. However, this study primarily focuses on predicting departure times for vehicles traveling straight through intersections. Therefore, the trajectory data used in this analysis predominantly originates from the through lanes. The actual scenes and corresponding datasets are depicted in Figure 3.

The intersections experienced heavy traffic but were undersaturated during data collection. To extract trajectory data, we developed a vehicle identification and tracking algorithm based on the OpenCV software library (OpenCV 3.3). Subsequently, we extracted vehicle trajectories at a sampling frequency of 10 Hz (equivalent to 0.1 s intervals) within 220 m from the stop line, capturing the vehicle index, lateral and longitudinal coordinates, vehicle length, and related information. To mitigate data noise arising from vehicle trajectory tracking in the video, we applied a Savitzky–Golay filter to smooth the vehicle velocity trajectory using a 2.5 s sliding time window. Using the feature and label extraction algorithm described in Section 4.3, we extracted approximately 38,890 valid samples from the collected UAV trajectory data. This dataset will be utilized for model training and analysis in the following sections.

In addition, the batch extraction of features and labels needs to consider lane-changing events. This study adopts the lane-changing recognition method proposed by Zhang [44], based on trajectory data. Specifically, a vehicle is deemed to be changing lanes into or out of the current lane when its entire body crosses the lane dividing line.

### 5.2. Applicability Test of GPR

**Figure 4:**
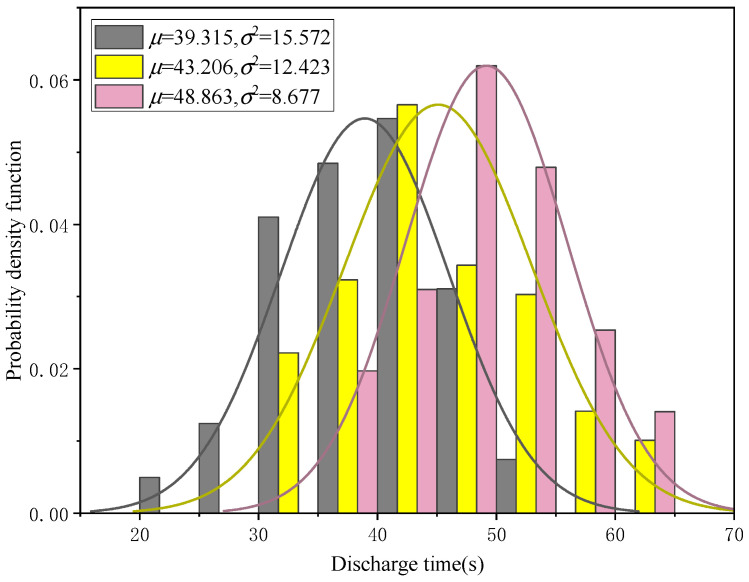
The probability density function of the observed departure times from three random datasets.

To further analyze the distribution of DT(*t*), and verify the applicability of GPR in vehicle departure time prediction, we extracted more than 584 vehicle trajectories in 34 consecutive signal cycles from the first intersection (dataset 1). The trajectories were then projected to the same signal cycle. Then, three sub-datasets were randomly selected at three moments during the red signal period (the remaining red light times corresponding to the three moments were 6 s, 15 s and 20 s). Figure 4 shows the probability density function of the observed departure times of vehicles from the three sub-datasets within the control range (*L* = 220 m). The Shapiro–Wilk (S-W) normality test was conducted, which confirmed the hypothesis that the departure time DT(*t*) from each dataset followed a normal distribution. Therefore, the application of GPR to the prediction of departure time under different traffic conditions is possible.

Similar properties have also been observed in other datasets (from two other signalized intersections). However, further discussion on this topic will not be provided here to maintain conciseness.

### 5.3. Key Feature Selection

**Figure 5:**
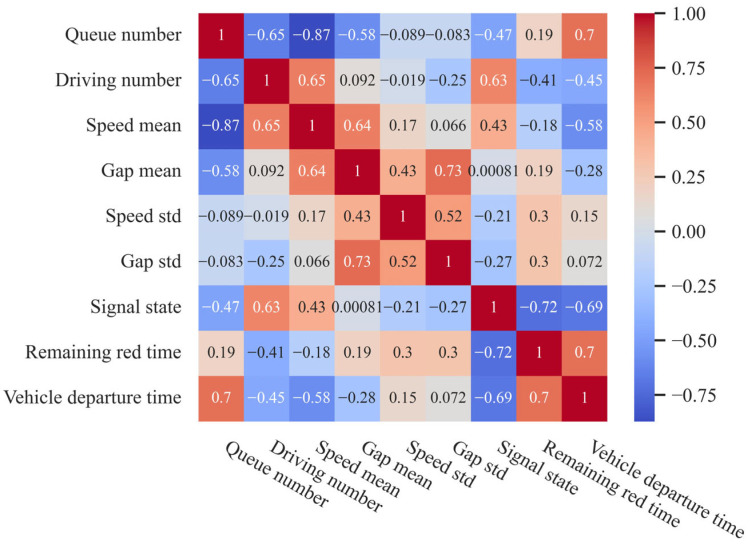
Pearson correlation coefficient matrix of features and target variable.

**Figure 6:**
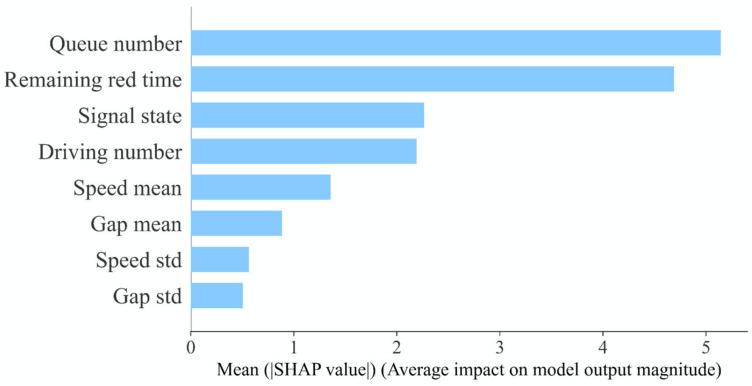
Feature importance rank based on SHAP values.

Based on the dataset extracted in Section 5.1, more than 7000 input (i.e., the feature variables listed in Table 2) and output pairs (i.e., the vehicle departure time) are used to calculate the Pearson correlation coefficient and SHAP values to identify key features. Figure 5 shows that the queue number, remaining red time, signal state, speed mean and driving number have stronger correlations with the vehicle departure time, and the correlation coefficients are 0.7, 0.7, −0.69, −0.58, and −0.45, respectively. Figure 6 shows that the importance rank of the features is consistent overall with the results of the Pearson correlation coefficient. The only difference is that the importance of the driving number is higher than that of the speed mean. Hence, as a trade-off between computational load and prediction accuracy, the five features mentioned above were ultimately selected as the inputs of the GPR-based vehicle departure time prediction model, i.e., $x\in {\mathbb{R}}^{5}$.

### 5.4. Model Training and Evaluation

#### 5.4.1. Training

In this section, we use the valid samples extracted in Section 5.1 under different scenarios covering the process of queue formation and dissipation. The random 70/30 split is adopted to evaluate the model’s generalization to unseen samples from three intersections (e.g., different time periods, traffic flow variations, and signal timing plans). This is a necessary first step before considering transfer to different intersections. The split does not aim to assess cross-intersection generalization, which would require a leave-one-intersection-out validation. The rationale is that the model is intended to be deployed at the same intersections where it was trained (or at intersections with nearly identical geometric and signal characteristics), and the random split mimics the scenario of encountering new traffic events at those same sites. We explicitly acknowledge that extrapolating to entirely new intersections without retraining remains an open question and is left for future work (see Section 6).

The training dataset was used to fit the GPR-based departure time prediction model, and the test dataset was used to evaluate model performance. During the model training process, GPstuff was used for hyperparameter inference [45]. Due to the absence of specific prior knowledge, the hyperparameters were given a broad gamma prior G(1, 100) [37]. Gradient-based optimization was used to maximize the log posterior probability, thereby obtaining the optimal hyperparameters with $\sigma_{m}=[2.599;\;2.361;\;3.125;\;37.906;\;5.409]$ corresponding to the five features, $\sigma_{f}=67.13$ and $\sigma_{\varepsilon }=1.46$. It is noted that these parameters should be updated if the traffic environment changes.

#### 5.4.2. Evaluation Metric

The mean absolute prediction error (MAPE), Root Mean Squared Error (RMSE), and $R^{2}$ (R-squared) are used to evaluate the prediction accuracy/performance of the DT as follows:

(10) $$\mathrm{MAPE}=\frac{1}{n}\sum_{i=1}^{n}\left|\frac{\hat{y_{i}}-y_{i}}{y_{i}}\right|,$$

(11) $$\mathrm{RMSE}=\sqrt{\frac{1}{n}\sum_{i=1}^{n}{\left(y_{i}-\hat{y_{i}}\right)}^{2}},$$

(12) $$R^{2}=1-\frac{\sum_{i=1}^{n}{\left(y_{i}-\hat{y_{i}}\right)}^{2}}{\sum_{i=1}^{n}{\left({\bar{y}}_{i}-y_{i}\right)}^{2}},$$

where $\hat{y_{i}}$ is the predicted DT for sample *i*, $y_{i}$ is the observed DT for sample *i*, ${\bar{y}}_{i}$ denotes the average value of observations, and *n* is the total number of samples in the test dataset.

#### 5.4.3. Results

**Figure 7:**
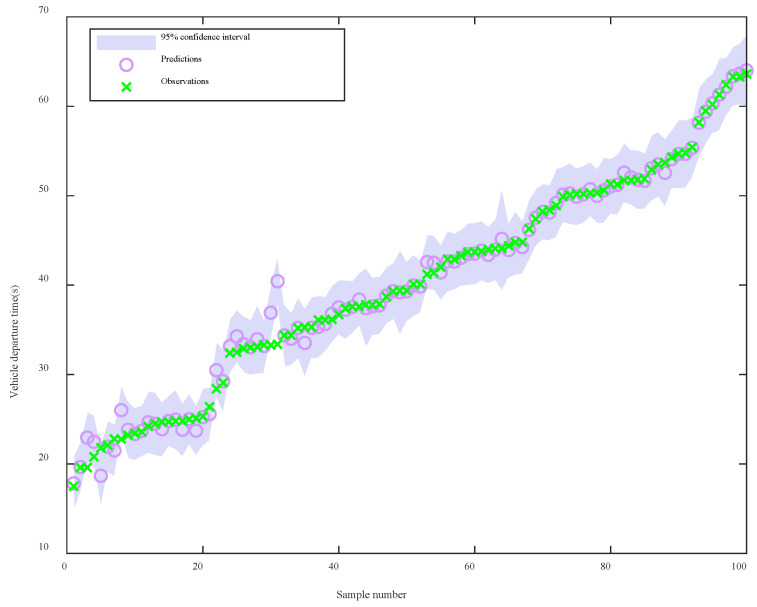
A comparison between the prediction of departure time with a 95% confidence level and the observations.

In this study, a GPR model was employed not only to predict the mean values but also to generate confidence intervals, facilitating a comprehensive evaluation of the predictive performance of the model. On the test dataset, our method achieves notably high accuracy with a mean absolute percentage error (MAPE) of 1.87%. One hundred samples were randomly selected, as illustrated in Figure 7, and the vast majority of observed departure time values fall within the 95% confidence interval, indicating the model’s credible prediction accuracy and stability. Despite a few outliers with significant deviations between predicted and actual values, the overall performance of the proposed model demonstrates reliability and effectiveness.

#### 5.4.4. Ablation Study on Feature Necessity

To empirically assess whether the observed correlations among predictors lead to functional redundancy, we conducted an ablation study. Pairwise correlation analysis reveals that queue_number and speed_mean are strongly negatively correlated (r = −0.87), while driving_number and speed_mean are moderately positively correlated (r = 0.65). To determine whether any feature can be safely removed without sacrificing predictive performance, we removed each candidate feature—queue_number, speed_mean, and driving_number—one at a time and retrained the GPR model under identical conditions (70/30 random split, same kernel, same hyperparameter inference). Performance was evaluated on both the test set (30% of the original data) and an independent validation set (100 new samples not used in training or testing).

**Figure 8:**
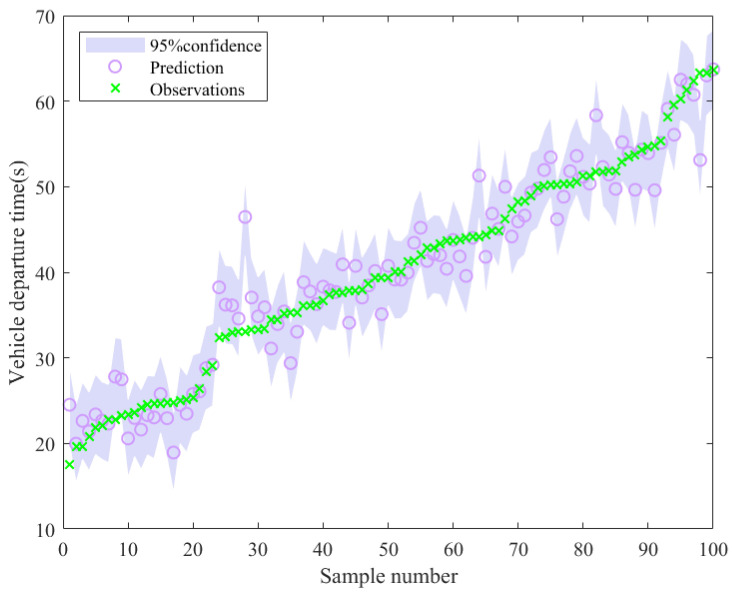
The prediction results without the queue_number feature (100 validation samples).

**Figure 9:**
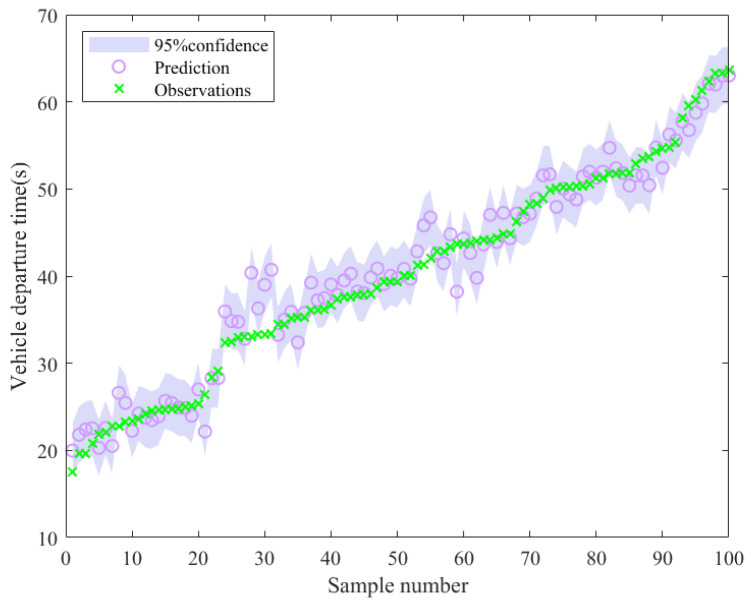
The prediction results after removing the average speed feature (100 validation samples).

**Figure 10:**
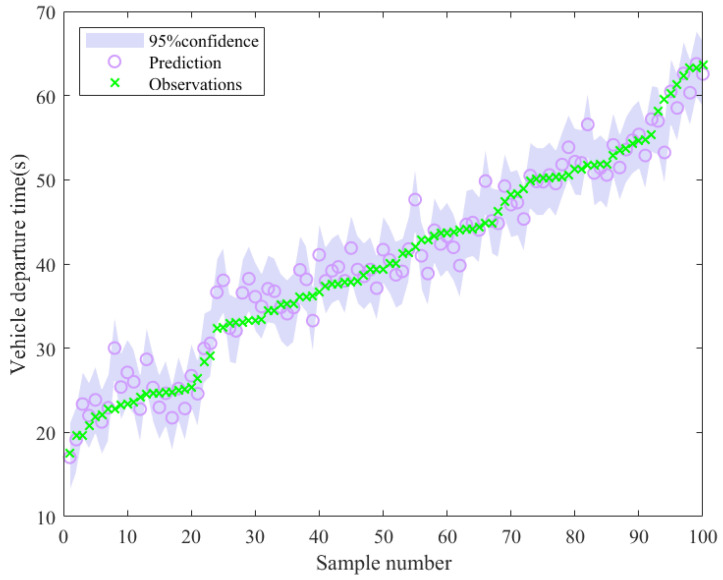
The prediction results after removing the driving number feature (100 validation samples).

Table 3 summarizes the performance metrics for the full model and each ablation case. The corresponding scatter plots (actual vs. predicted) for the validation set are presented in Figure 8, Figure 9 and Figure 10. Removing any single feature causes a substantial drop in prediction accuracy. On the test set, the MAPE increases by a factor of 2–3 (from 1.87% to 4.28–5.35%), and degradation is even more pronounced on the independent validation set (e.g., MAPE rises to 6.15% when queue_number is removed). All three metrics (R^2^, MAPE, RMSE) consistently worsen, indicating that the effect is robust and not due to random variation. Crucially, strong correlation does not imply redundancy. Despite the high negative correlation (−0.87) between queue_number and speed_mean, removing either one significantly harms performance. The same holds for the moderate positive correlation (0.65) between driving_number and speed_mean. This demonstrates that each feature captures distinct, complementary aspects of the queue dissipation process that the others cannot compensate for.

### 5.5. Comparative Analysis

We also implemented four regression models (e.g., linear regression, decision tree, MLP-NN, and XGBoost).

***Linear regression***: Linear regression assumes a direct proportionality between predictors and the response, allowing for the straightforward interpretation of coefficients. It performs the best when the relationship between variables is indeed linear, while its inability to capture nonlinear relationships can lead to inaccurate predictions in complex datasets.

***Decision trees***: Decision trees utilize a tree-like structure to represent decision-making processes, making them intuitive and easy to understand. The data is recursively split based on conditional tests on input features, resulting in a set of rules that lead to predictions. However, they are prone to overfitting, especially on noisy or complex datasets, and may suffer from high variance due to sensitivity to specific splits chosen during training.

***Multilayer perceptron neural networks***: MLP-NNs are composed of multiple interconnected layers of neurons arranged in a feedforward manner. They excel at capturing nonlinear relationships in the data through nonlinear transformations and activation functions, enabling them to model complex patterns and relationships. This makes them particularly suitable for handling high-dimensional input spaces and generalizing well to unseen data. However, training MLP-NNs can be computationally expensive, especially on large datasets.

***XGBoost***: XGBoost, a gradient boosting library, constructs an ensemble of decision trees to achieve highly accurate predictions. It leverages various regularization techniques and optimization strategies to boost its performance, making it a powerful tool for predictive analytics. XGBoost stands out for its scalability, performing exceptionally well even on large datasets. Furthermore, it gracefully handles missing values and includes built-in cross-validation, facilitating model selection.

Given that machine learning models are susceptible to overfitting, the models employed in this study are meticulously selected and employed. Furthermore, the utilization of the *k*-fold validation technique serves as a robust method, thoroughly evaluating the models’ capacity to generalize across diverse data segments while effectively minimizing the likelihood of overfitting. Note that several configurations of hyperparameters were tested for all the models, and the best-performing configuration (in terms of the highest accuracy) is reported in Table 4. Unlike linear regression, which provides explicit physical interpretations through its coefficients, the Gaussian process regression model does not directly reveal the physical relationship between input features and the tail vehicle departure time, as it is a nonparametric probabilistic method that focuses on predictive accuracy and uncertainty quantification.

Table 5 illustrates the predictive performance of various machine learning models on the same test dataset. It is observed that linear regression and decision trees show relatively poorer performance, indicated by higher MAPE and RMSE values, as well as lower R^2^ scores. This could be attributed to their limited capability in capturing nonlinear relationships within the data. Conversely, MLP-NN and XGBoost demonstrate superior performance, showcasing the effectiveness of neural networks and gradient boosting in managing intricate datasets. However, Gaussian process regression (GPR) surpasses all other models by achieving the lowest MAPE and RMSE, along with the highest R^2^. Additionally, GPR’s MAPE is reduced by 5.146% approximately on average, with an RMSE reduction of 2.219 and R2 increase of 0.091, compared to other models. This outcome aligns with GPR’s recognized characteristics, including its nonparametric nature, capacity to handle uncertainty, and underlying assumption of smoothness. These attributes empower GPR to adapt flexibly to diverse data distributions and capture complex relationships, ultimately leading to its exceptional performance.

**Figure 11:**
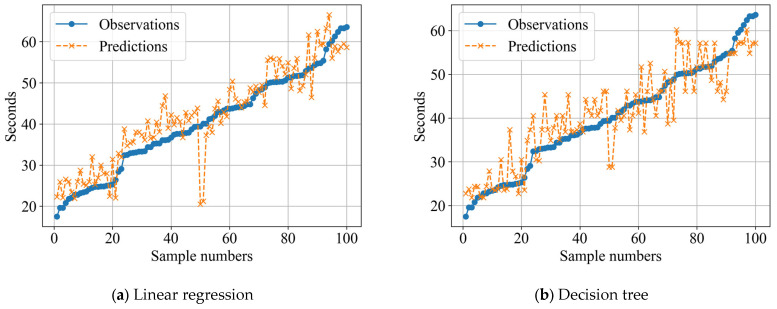

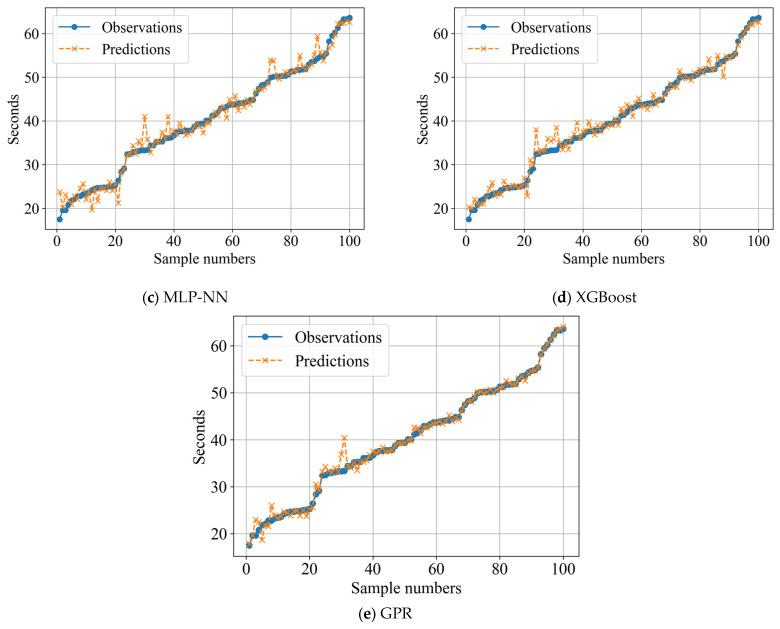
The prediction results of the five models.

Figure 11 presents the vehicle departure time prediction results (randomly selected 100 test samples) based on various machine learning models. In this figure, the blue line represents the actual departure times of vehicles, while the yellow dashed line represents the predictive ones. We observe that the vehicle departure time predictions generated by GPR (Figure 11e) align more closely with the actual departure times of vehicles.

### 5.6. Robustness Evaluation

To assess the robustness of the model, we introduced random noise into the test data, simulating sensor detection errors caused by external uncertainties in real-world traffic environments. We generated random noise data using uniform distribution functions. Specifically, on the original dataset, we added random noise as follows: ±1 m/s to the feature of speed mean, ±0.5 m to the gap mean, ±1 m/s to the speed std, and ±1.5 m to the gap std. After applying several trained models for prediction, we obtained the evaluation results shown in Table 6. It is evident that the predictive performance of these models decreased in the presence of noise compared to Table 5, with XGBoost demonstrating the best performance. This could be attributed to the fact that GPR models consider the global structure of the data and attempt to find a function that explains the entire dataset. When there are local noises or outliers in the data, GPR may struggle to effectively handle them. On the other hand, XGBoost constructs multiple decision trees to capture the local structure of the data and can prevent overfitting during training through techniques like pruning. Therefore, XGBoost may have a competitive advantage and better robustness in handling data with local noise. Thus, when selecting a model, it is essential to consider both the accuracy of traffic parameter detection and the distribution of data noise. Specifically, if the detection accuracy of traffic parameters (model input features) is high, choosing the GPR model may yield better predictive results. However, when there is certain degree of random noise in the detection of traffic parameters, the predictive performance of the XGBoost model may be more reliable and preferable.

## 6. Conclusions

We introduce a novel method for predicting the departure time of the tail vehicle within a fleet near signalized intersections using Gaussian process regression (GPR). This method effectively addresses traffic uncertainties during queue formation and dissipation. Under optimal data conditions, our method exhibits superior prediction accuracy and stability compared to other machine learning algorithms, leveraging the inherent Gaussian process distribution characteristics of vehicle departure times within a specified spatial range. On the test dataset (random 70/30 split from three intersections), our method achieves notably high accuracy with a mean absolute percentage error (MAPE) of 1.87%, RMSE of 1.130, and R-squared value (R^2^) of 0.991. When compared to linear regression, decision trees, multilayer perceptron neural networks, and the XGBoost model, our approach demonstrates an average reduction in the MAPE of 5.146%, an RMSE reduction of 2.219, and an R^2^ increase of 0.091. Additionally, comparative experiments using simulated noise data reveal that while the robustness of the GPR model is slightly inferior to the XGBoost model, it outperforms other models. This underscores the importance of selecting appropriate prediction models based on real sensor perception and detection errors for practical applications, thereby contributing to the evaluation and standardization of traffic parameter sensing accuracy. Importantly, our method requires the input of macroscopic traffic parameters such as queued vehicle count and average speed, which are readily available in intelligent and connected traffic environments, enhancing its practicality.

The above results are derived from data collected at three specific multi-lane signalized intersections under a random 70/30 split. Therefore, the conclusions are strictly valid for the three typical intersections (or for intersections with highly similar geometric and signal control characteristics after retraining). The current validation strategy does not support the claim that the model can be directly applied to unseen intersections without retraining or adaptation. This is a deliberate and transparent limitation: our core contribution is to demonstrate that GPR can effectively learn queue dissipation patterns within a given intersection under varying traffic conditions over time, rather than to assert cross-site transferability. Accordingly, we do not offer a ready-to-deploy universal model for any intersection but rather a modeling framework and feature set that can be reused or retrained for other sites when appropriate local data are available. For a truly generalizable model, a cross-intersection validation (e.g., training on some intersections and testing on others) would be required, which we plan as future work. Additionally, we will explore deep learning architectures (e.g., Graph Neural Networks for spatial interactions across lanes, Transformers for temporal dependencies) when larger-scale data become available. Finally, we will enhance the proposed prediction model to develop a complete eco-driving trajectory planning system for connected vehicles, including corresponding software and hardware components.

## Figures and Tables

**Table 1:** Summary of existing studies and research gaps in queue-related prediction at signalized intersections.

Research Area	Representative Studies	Methodology/Data	Key Limitation/Unresolved Issue
Queue dissipation characteristics	Stephanopoulos [7]; Zhuang-zhi et al. [8]; An et al. [9]; Bhattacharyya et al. [10]; Al-kaissi et al. [11]; Mondal & Sharma [12]; Sharma et al. [13]; Dey et al. [15]; Mondal et al. [16]; Qu et al. [17]; Srivastava et al. [2]; Wei Xu et al. [18]	Headway distribution models, HMMs, vehicle passing interval, shockwave theory, improved CTM, optimal velocity model	Focus on macroscopic or steady-state flow; rarely predict individual vehicle departure time; most assume fully formed stationary queues
Queue length estimation	Bezuidenhout et al. [19]; Yu et al. [20]; Zhan et al. [4]; Mei et al. [3]; recent V2X/lidar studies [21,22]	Loop detector data, LWR shockwave theory, Gaussian process, GPS trajectories, Bayesian estimation	Estimate queue length, not departure time; often require dense detector data or assume homogeneous arrival/departure
Estimation of other traffic parameters (delay, speed, travel time)	Teng et al. [23]; Lu et al. [24]; Sun et al. [25]; Guchang et al. [26]; Fang et al. [27]	GPS data, shockwave-based delay model, PW model, wavelet–Elman neural network	Target delay or speed rather than last vehicle departure time; many are single-lane or post-evaluation only
Eco-driving considering queue constraints	Xin et al. [28]; Chao Sun et al. [29]; Fei Ye et al. [30]; Hao Yang et al. [31]; Dong [32,33]	Predictive IDM, robust optimization, EAD strategy, shockwave-based last vehicle time, kinematic models	Consider queue dissipation time or last vehicle departure but rely on homogeneous flow or stationary queue assumptions; not designed for uncertain/random arrival scenarios
Direct prediction of queue dissipation time/last vehicle departure	Linear regression [34]; Chen et al. (deep learning) [6]	Linear regression with traffic composition variables; deep learning from video	Applicable only when a stationary queue exists after green onset; not for undersaturated or randomly forming queues

**Table 2:** Definitions of features.

Features	Type	Descriptions
Queue number	Discrete	Number of queueing vehicles with velocity lower than 1 m/s.
Driving number	Discrete	Number of moving vehicles with velocity higher than 1 m/s.
Speed mean	Continuous	Average velocity of queueing and moving vehicles.
Gap mean	Continuous	Average gap of queueing and moving vehicles.
Speed std	Continuous	Velocity standard deviation of queueing and moving vehicles.
Gap std	Continuous	Gap standard deviation of queueing and moving vehicles.
Signal state	Discrete	Signal state when a subject CAV reaches the control boundary, red signal set 0, green set 1; amber is regarded as green.
Remaining red time	Continuous	Remaining red time from the current moment to the onset of the next green signal (the value is set to 0 during green signals).

**Table 3:** Ablation experiment results.

Removed Feature	Test Set (R^2^/MAPE/RMSE)	Validation Set (R^2^/MAPE/RMSE)
None (full model)	0.991/1.87%/1.130	(consistent best performance)
queue_num	0.9582/5.35%/2.360	0.9312/6.15%/3.114
speed_mean	0.9784/4.28%/1.695	0.9656/4.64%/2.201
driving_num	0.9689/5.13%/2.035	0.9590/5.20%/2.404

**Table 4:** The configurations of the comparative machine learning models adopted in this study.

Model	Hyperparameters Selected
Linear regression	The coefficients of queue number, driving number, speed mean, signal state, and remaining red time are 2.549, 1.449, −0.248, −2.049, 0.967, respectively, and the constant term is 5.859.
Decision tree	Max_depth: 10.0; min_samples_leaf: 0.01; min_samples_split: 0.01; and other parameters keep the default settings.
MLP-NN	Activation: tanh; optimizer: Adam; alpha: 0.01; beta 1: 0.9; beta 2: 0.999; epsilon: 1 × 10^−8^; hidden_layer_sizes: 64, 64, 32, 32; learning_rate_init: 0.001; max_iter: 100; random state: 123; and other parameters keep the default settings.
XGBoost	Objective: squared error; base score: 0.5; booster: gbtree; gamma: 0.8; learning_rate: 0.08; max_bin: 256; max_cat_to_onehot: 4; max depth: 8; min child weight: 4; n_estimators: 600; random state: 27; regularization alpha: 0.001; regularization _lambda: 0.1; sampling_method: uniform; scale_pos_weight: 1; subsample: 0.8; scoring: negative mean squared error; seed: 27; and other parameters keep the default settings.
GPR	Kernel function: ARD squared exponential; $\sigma_{m}=[2.599;\;2.361;\;3.125;\;37.906;\;5.409];$ $\sigma_{f}=67.129;$ $\sigma_{\varepsilon }=1.458.$

**Table 5:** MAPE and RMSE comparison between different models.

Model	MAPE/%	RMSE	R^2^
Linear regression	10.688	4.893	0.830
Decision tree	10.851	5.178	0.810
MLP-NN	3.582	1.863	0.975
XGBoost	2.963	1.462	0.985
GPR	1.875	1.130	0.991

**Table 6:** MAPE and RMSE comparison between different models with noisy data.

Model	MAPE/%	RMSE	R^2^
Linear regression	10.698	4.886	0.831
Decision tree	11.245	5.355	0.796
MLP-NN	9.412	5.157	0.817
XGBoost	5.894	2.905	0.940
GPR	9.055	4.995	0.823

## Data Availability

The raw data supporting the conclusions of this article will be made available by the authors on request.
